# Uncovering the Genetic Landscape of Spinal Dysraphism: A Retrospective Analysis of 150 Fetal Cases

**DOI:** 10.1002/pd.70037

**Published:** 2025-12-12

**Authors:** I. Bedei, A. Feresin, R. Zemet, D. Berner, D. Polidori, K. Fröbius, A. Skakkebæk, R. Axt‐Fliedner, M. Shoukier, C. Keil

**Affiliations:** ^1^ Department of Prenatal Medicine and Fetal Therapy Justus‐Liebig University Giessen Giessen Germany; ^2^ Department of Molecular Medicine Aarhus University Hospital Aarhus Denmark; ^3^ Independent Researcher Italy; ^4^ Department of Obstetrics and Gynecology Baylor College of Medicine Houston Texas USA; ^5^ Eurofins Humangenetik und Pränatal‐Medizin MVZ GmbH Munich Germany; ^6^ Department of Obstetrics and Gynaecology Hospital San Pietro Fatebenefratelli Tor Vergata University of Rome Rome Italy; ^7^ Department of Human Genetics Justus‐Liebig University Giessen Germany; ^8^ Department of Clinical Genetics Aarhus University Hospital Aarhus Denmark; ^9^ Department of Clinical Medicine Aarhus University Aarhus Denmark; ^10^ Department of Prenatal Medicine and Fetal Therapy Philipps University Marburg Germany

**Keywords:** chromosomal microarray (CMA), fetal surgery, myelomeningocele, myeloschisis, neural tube defect, spina bifida, spinal dysraphism (SD), trio exome sequencing (ES)

## Abstract

**Objective:**

Spinal dysraphism (SD) results from incomplete neural tube closure and encompasses a heterogeneous group of congenital anomalies with genetic and environmental etiologies. Although genetic contributions are recognized, causative variants remain insufficiently defined, and the clinical implications of extended genetic testing on parental decision‐making are not well characterized. This study evaluated the diagnostic utility of extended genetic testing, including exome sequencing (ES), in fetuses with prenatally diagnosed SD and its influence on clinical management.

**Methods:**

We retrospectively analyzed 150 pregnancies with a prenatal diagnosis of SD referred to our center between July 2021 and May 2025. All cases underwent detailed phenotyping, genetic counseling, and were offered extended genetic testing, including karyotyping, chromosomal microarray (CMA), and trio‐based ES.

**Results:**

Genetic testing, including karyotyping (110/110), CMA (61, 55.5%), and ES (66, 60.0%), was performed in 110 fetuses. Genetic anomalies were detected in 19 fetuses (17.3%). ES revealed or confirmed 16 pathogenic, likely pathogenic, or uncertain variants in 14/66 (21.21%) fetuses, including one with three distinct variants. Notably, twelve of these fetuses would not have been identified without ES. Although no definitive causative molecular variants were detected, ES results influenced the parental decision to terminate the pregnancy in four cases.

**Conclusion:**

ES increases diagnostic yield in SD and may influence prenatal decision‐making.

## Bulleted Statements

- What is already known about the topic?
  ◦ Exome sequencing (ES) has demonstrated an increased diagnostic yield in fetuses with structural anomalies, including central nervous system malformations. Its role in spinal dysraphism (SD), particularly in the context of prenatal surgery, has not been established.
- What does this study add?
  ◦ ES offers a higher diagnostic yield than karyotyping and chromosomal microarray in fetuses with SD. The turnaround time is compatible with the timeframe for prenatal surgical planning. ES findings have the potential to influence parental decision‐making.

## 1 Introduction

Neural tube defects (NTDs) are a heterogeneous group of severe congenital malformations arising from incomplete closure of the neural tube during early embryonic development. They encompass a spectrum of anomalies, including anencephaly, spinal dysraphism (SD), encephalocele, iniencephaly, and craniorachischisis [1]. NTDs may present as isolated malformations, occur in combination with other structural anomalies, or arise as part of broader genetic syndromes [2]. As one of the most common categories of birth defects, NTDs have a global prevalence of approximately 2 cases per 1000 births, translating to over 200,000 affected pregnancies worldwide annually [1, 3]. These defects are associated with significant perinatal and childhood morbidity and mortality, long‐term disability, and substantial healthcare costs [2, 4]. Early and accurate prenatal diagnosis and identification of the underlying etiology are critical.

Although most NTDs are thought to arise from multifactorial etiologies—including environmental and nutritional influences such as folate deficiency—there is increasing recognition of a substantial genetic contribution, particularly in cases associated with additional congenital anomalies [2, 5, 6]. NTDs have an estimated heritability of up to 60%, and chromosomal abnormalities have historically been the most commonly identified genetic findings in prenatal cases [7]. Trisomy 13, trisomy 18, and triploidy are most frequently observed, particularly in non‐isolated NTDs, where chromosomal abnormalities are identified in up to 25% of cases, compared to 2.6% in isolated cases [8, 9, 10, 11, 12, 13]. Select microdeletions and microduplications have also been implicated [14, 15, 16, 17]. However, the role of monogenic disorders in NTDs remains incompletely defined. While over 250 genes causing NTDs in mice have been identified through Mendelian segregation studies, the genetic basis of human NTDs remains poorly understood [18, 19]. Neurulation involves numerous molecular and cellular processes, and variants in genes regulating folate metabolism, planar cell polarity (e.g., Wnt signaling), ciliary function, and major developmental signaling pathways such as the HOX gene family, sonic hedgehog, and protein kinase A have been implicated in both syndromic and nonsyndromic NTDs [20, 21, 22, 23, 24, 25, 26, 27]. Building on these findings, exome sequencing (ES) studies in familial NTD cases have identified novel candidate genes such as *MTHFR*, *DLC1*, *ITGB1*, and *FZD6* [28, 29]. Nevertheless, despite substantial insights from animal models, the contribution of human genetic variation to NTDs has yet to be fully elucidated.

Prenatal genetic evaluation of NTDs has traditionally relied on karyotyping, particularly in the context of fetal surgery eligibility as defined by the Management of Myelomeningocele Study (MOMS), which requires a normal chromosomal analysis [30]. More recently, and in line with recommendations from ACOG and other professional societies, chromosomal microarray analysis (CMA) has become the standard first‐tier test for pregnancies with prenatally detected major congenital anomalies, including NTDs [2, 6, 31]. While karyotype and CMA can detect clinically significant chromosomal abnormalities—especially in cases with multiple anomalies—their combined diagnostic yield for congenital anomalies is approximately 40% [32, 33, 34, 35]. As these approaches do not detect monogenic disorders, most affected pregnancies remain without a definitive genetic diagnosis. This limitation has prompted increasing interest in next‐generation sequencing technologies, particularly Exome sequencing (ES), which has emerged as a powerful diagnostic tool for structural anomalies. A recent systematic review by Mellis et al. demonstrated that prenatal trio ES provides a diagnostic yield of 31% overall, increasing to 42% in pre‐selected cases with a higher a priori risk for monogenic disease [36]. Reflecting this shift in clinical practice, the most recent committee opinion from the International Society for Prenatal Diagnosis (ISPD) supports the use of prenatal trio ES for fetuses with any unexplained congenital anomaly [37].

Despite increasing recognition of the genetic heterogeneity underlying NTDs, the contribution of rare, single‐gene variants, particularly in fetuses with prenatally diagnosed NTDs, remains poorly defined. While ES has shown utility in identifying pathogenic variants in various congenital anomalies, limited data are guiding its application and interpretation in cases of spinal dysraphism detected during prenatal imaging. This knowledge gap is particularly significant given the clinical importance of establishing an etiology to inform parental counseling, guide prenatal and neonatal management, and refine recurrence risk assessment. In this study, we aimed to evaluate the diagnostic utility of extended genetic testing with a focus on ES in a cohort of fetuses with prenatally diagnosed open and closed SD to elucidate potential genetic contributions to these distinct subtypes. Additionally, we sought to assess whether the identification of (likely) pathogenic variant impacts parental decision‐making during the prenatal period.

## 2 Methods

This retrospective, single‐center study included data from 150 pregnancies with a confirmed prenatal diagnosis of fetal spinal dysraphism, all of which were evaluated, counseled, and/or treated at the University Hospital Gießen and Marburg between July 2021 and May 2025. The center is a national referral unit specializing in prenatal surgical interventions for fetuses with open spinal dysraphism.

### 2.1 Study Population

Fetuses diagnosed with either open or closed spinal dysraphism were included in the study. Invasive genetic testing was systematically offered to all patients. For those considering prenatal fetal surgery, an invasive genetic diagnosis was a prerequisite. Fetuses presenting minor/moderate anomalies not encompassed by the original MOMS inclusion criteria were not excluded a priori. Following interdisciplinary case‐by‐case evaluation involving geneticists, fetal medicine specialists, pediatric neurologists, pediatric neurosurgeons, and neonatologists, fetal surgery was offered if the anomalies were deemed clinically non‐severe and unlikely to impact perioperative management or neonatal outcome. This approach represents a deliberate deviation from the strict MOMS protocol, justified by institutional consensus and ethical considerations. Fetuses with major anomalies were excluded. Ethical approval for this study was obtained from the Ethics Committees of Justus‐Liebig‐University, Gießen (IRB approval AZ 161/20), and Philipps University Marburg (IRB approval AZ 23–280 BO).

Because of the retrospective nature of this study, informed consent was waived, as only anonymized clinical data were analyzed.

### 2.2 Clinical and Genetic Evaluation

A standardized evaluation protocol was implemented for all patients, aligning with the guidelines established by the Management of Myelomeningocele Study (MOMS) [30]. A comprehensive fetal anatomical assessment by ultrasound and MRI was performed to evaluate both structural malformations associated with spinal dysraphism and additional malformations identified during initial ultrasound examinations, regardless of presumed association. Genetic testing was primarily performed on samples obtained through amniocentesis (AC). DNA was extracted from amniotic fluid, and maternal cell contamination was excluded in all cases before analysis. Routine invasive genetic testing, including conventional karyotyping, chromosomal microarray analysis (CMA), and exome sequencing (ES), was offered to all patients presenting for evaluation at our center. In Germany, conventional karyotyping remains the first‐line cytogenetic diagnostic modality in prenatal settings. Chromosomal microarray analysis is not routinely reimbursed by public health insurance and therefore requires explicit justification and prior approval for cost coverage. Following current international recommendations, a trio‐based genetic analysis approach—comprising the index fetus and both biological parents—was implemented as the standard diagnostic strategy [37].This approach was selected to optimize variant interpretation and enable the detection of de novo mutations, compound heterozygosity, and inheritance patterns. Deviations from the trio‐based design occurred in a limited number of cases, specifically when biological paternal samples were unavailable (e.g., due to non‐participation or untraceability) or when reimbursement for parental testing was restricted by health insurance limitations. These exceptions were managed on a case‐by‐case basis, with diagnostic testing adapted accordingly (e.g., proband‐only or duo analyses). For all cases in which pathogenic or likely pathogenic findings were identified, the applied testing constellation (trio, duo, or proband‐only) was explicitly annotated in the corresponding result table to ensure transparency and reproducibility of variant interpretation (Table 3). Variant interpretation of ES findings was performed in accordance with the guidelines of the American College of Medical Genetics and Genomics (ACMG) [38]. Variants were classified into five categories: pathogenic, likely pathogenic, variant of uncertain significance (VUS), likely benign, and benign. In prenatal genetics, class 3 variants are generally not disclosed because limited fetal phenotyping and uncertain clinical significance may lead to parental anxiety or misinterpretation in counseling. However, within this research framework, we reported selected VUS after multidisciplinary review when the implicated genes represented strong biological candidates for NTD pathogenesis, and the parents had explicitly consented to receive potentially relevant but unconfirmed findings. Pre and post‐test counseling was conducted by specialists in fetal medicine with certified qualifications in human genetics, following national requirements, and/or by board‐certified clinical geneticists. Counseling explicitly addressed the possibility of detecting secondary findings in the fetus or parents, unrelated to the primary indication of spinal dysraphism. In cases of abnormal results, post‐test counseling was provided by an expert in medical genetics.

### 2.3 Karyotype Analysis of Amniotic Fluid Cells

Amniotic fluid cells were cultured according to standard protocols. Cells were harvested, fixed and prepared for microscopic analysis. After GTG‐staining, the metaphases were analyzed and evaluated using the Axioscope 5 POL light microscope and evaluated with the metasystem's software Ikaros 6.3.1.

### 2.4 Chromosomal Microarray Analysis (CMA)

CMA was performed using the Illumina HumanCytoSNP‐12v2.1 SNP array on an iScan system (Illumina Inc.). The protocol included DNA amplification, hybridization, fluorescence labeling, and data acquisition via KaryoStudio v1.4 software. Genotype and copy number variation analyses were based on B‐allele frequency and log2 ratio assessments, with Genome Build GRCh37 as the reference.

### 2.5 Exome Sequencing (ES)

Genomic DNA was extracted from EDTA blood and amniocytes using the QIAamp DNA Blood Mini Kit (Qiagen, Hilden, Germany) according to the manufacturer's protocol. DNA concentration was quantified with the Qubit system (dsDNA Assay Kit; Thermo Fisher Scientific, Waltham, MA, United States). ES was performed using the Illumina Twist Exome 2.0 Plus kit (Illumina, San Diego, CA, United States) following the manufacturer's guidelines. The hybrid capture method was employed to enrich and amplify target regions, with library preparation performed using the Illumina DNA Prep with Exome 2.0 Plus Enrichment Kit. Massively parallel sequencing was conducted on a NextSeq2000 Sequencing System (Illumina, San Diego, CA, United States). ES quality control criteria required a minimum coverage of 20 × for 97% of the target regions.

Variant selection/filtering and variant interpretation: Classification of the variants was based on the ACMG pathogenicity criteria using a 5‐tier classification system and with consideration of current recommendations from ACGS (Association for Clinical Genomic Science) and the SVI WG (Sequence Variant Interpretation Working Group) or VCEPs (Variant Curation Expert Panels) from ClinGen, if applicable [38].

### 2.6 Molecular Findings Interpretation

Considering the prenatal setting and the indication of in‐utero surgery for SD correction, abnormal results from karyotype, CMA, and ES have been comprehensively interpreted in the light of the test indication, diagnosis, prognosis determination, and case management. We then classified the reported abnormal findings into the following major categories according to their clinical role:

1. *Evidence‐based or possible association with SD*: VUS and LP/P findings with existing or partial evidence supporting an association with SD, including molecular data already reported in SD patients or having a potential contributive role in SD.
2. *Findings unrelated to SD and related to significant prognosis modification or clinical actionability*: LP/P findings regardless SD, associated with conditions with clinical severity/potential neurodevelopmental implications/prognosis worsening/with or without well‐established evidence‐based clinical management guidelines, available disease‐modifying treatments; chromosomal abnormalities (i.e., reciprocal translocation) regardless SD, associated with family reproductive counseling indicating familiar cascade testing.

### 2.7 Statistical Analysis

Analyses were conducted using the R Statistical language (version 4.3.2; R Core Team, 2023). Continuous variables were described using mean ± standard deviation and median and interquartile ranges. Differences were tested using the Wilcoxon Rank Sum test. Categorical parameters are reported as percentages for each respective groups and as absolute frequencies. Associations between categorical parameters were tested using Fisher's Exact test.

## 3 Results

### 3.1 Cohort Description and Lesion Subtypes

Between July 2021 and May 2025, 150 fetuses diagnosed with spinal dysraphism (SD) were evaluated. The cohort comprised 89 cases (59.3%) with myelomeningocele (MMC), 46 cases (30.7%) with myeloschisis (MS), and 15 cases (10%) presenting with closed or hybrid lesions. Fetuses with severe non‐associated anomalies were excluded from the study.

### 3.2 Maternal Characteristics

The mean maternal age across the cohort was 31.2 years (5.1), with no statistically significant difference between groups with and without genetic abnormalities. Mean gestational age at diagnosis was 22.1 (2.8) and median body mass index (BMI) was 26.6 (23.4–30.1), both of which were comparable between groups (Table 1).

**TABLE 1:** Demographic information.

	*N* (%)	Genetic testing normal	Genetic testing abnormal	No genetic testing	All 150 (%)	*p*‐value (genetic testing normal vs. not normal)
		*N* = 91	*N* = 19	*N* = 40		
Age (mean/SD)	150	31.1 ± 5.2	30.6 ± 4.6	31.7 ± 5.0	31.2 ± 5.1	0.66^a^
GA (mean/SD)	150	22.1 ± 2.5	21.6 ± 2.2	22.5 ± 3.6	22.1 ± 2.8	0.78^a^
BMI (median/IQR) (preconception)	150	26.5 (23.0–29.6)	25.8 (23.8–29.7)	27.7 (24.1–32.5)	26.6 (23.4–30.1)	0.76^a^
Lesion type						
– MMC	150	56 (61.5)	11 (57.9)	22 (55)	89 (59.3)	0.55^b^
– MS		28 (30.8)	5 (26.3)	13 (32.5)	46 (30.7)	
– CSD		7 (7.7)	3 (15.8)	5 (12.5)	15 (10.0)	
Not associated anomalies^c^	150	3 (3.3)	2 (10.5)	3 (7.5)	8 (5.3)	0.2^b^

*Note: x ± s* represents X ± 1 SD. a (b‐c) represents the median and interquartile range. Numbers after absolute frequencies represent proportions of the column.

Abbreviation: BMI, body mass index; CSD, closed spinal dysraphism; GA, gestational age; MMC, Myelomeningocele; MS, Myeloschisis.

^a^ Wilcoxon rank sum test.

^b^ Fisher's Exact Test for Count Data with simulated *p*‐value (based on 2000 replicates); only data for genetic testing was considered.

^c^ Hydronephrosis, Tricuspid Insufficiency, Lipoma of the Corpus callosum, Malalignment ventricular septum defect (VSD) and low‐set ears, muscular VSD, perianal appendage, singular umbilical artery, SUA.

### 3.3 Prenatal Genetic Testing Uptake and Modalities

**FIGURE 1:**
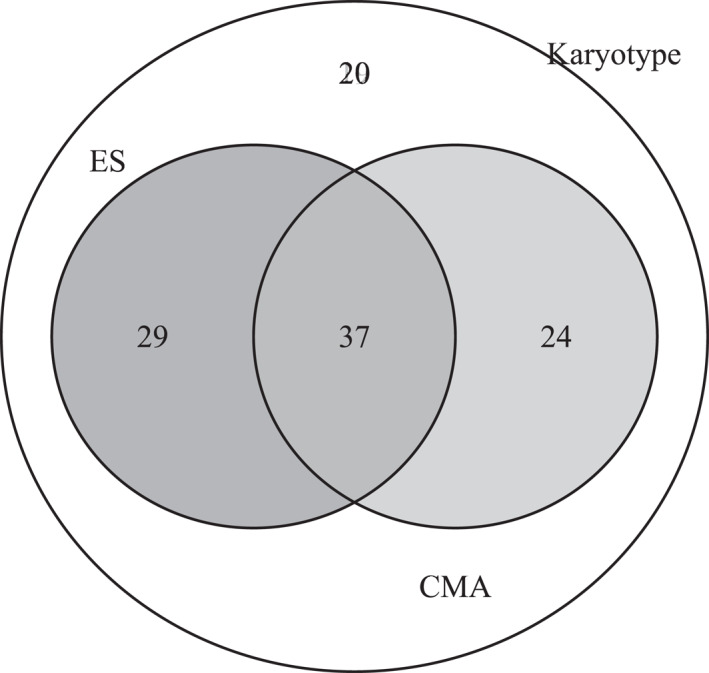
Distribution of distinct genetic testing modalities.

All patients were offered invasive prenatal genetic testing as part of standard clinical management. Overall, invasive genetic testing was performed in 110 fetuses (73.3%), while 40 patients (26.7%) declined any testing. Karyotyping was performed in all cases (110/110) as the baseline cytogenetic assay. CMA was conducted in 61/110 cases (55.5%), and ES was performed in 66 cases (60.0%). A total of 37 fetuses (33.6%) underwent all three genetic modalities (karyotype, CMA, and ES), and 20 had only a karyotype (Figure 1).

### 3.4 Genetic Findings

**FIGURE 2:**
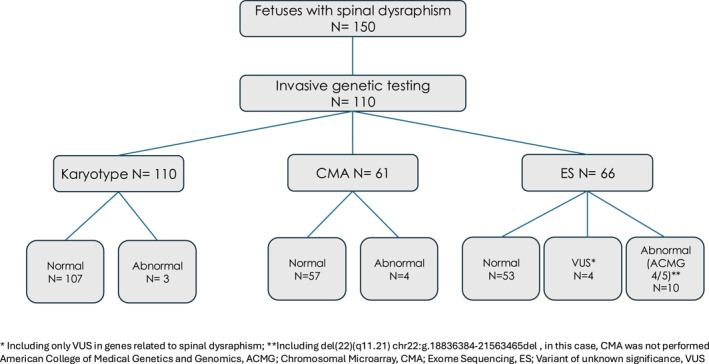
Flow chart.

**FIGURE 3:**
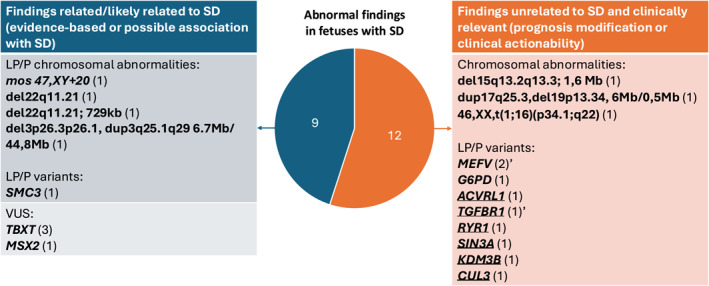
Interpretation of results according to the clinical role. The pie shows the distributions of (1) Findings related/likely related to SD (evidence‐based or possible association with SD) (in blue); (2) Findings unrelated to SD and clinically relevant (prognosis modification or clinical actionability) (in orange). On the left, the related table (in blue) lists LP/P chromosomal abnormalities, LP/P variants, and VUS reported as related or likely related to SD. On the right, the related table (in orange) lists chromosomal abnormalities and LP/P variants reported regardless of SD as clinically relevant. Cytogenomic results are simplified (details are presented in Table 1), and genes only are listed (variants are presented in Table 2). After each result, the number in brackets refers to the number of cases. LP, likely pathogenic; P, pathogenic; SD, spinal dysraphism; VUS, variant of uncertain significance. ‘: variants in the same case (Case 19). Underlined genes are included in the ACMG secondary finding list. Double‐underlined genes are associated with neurodevelopmental conditions.

Among the 110 fetuses tested, 19 (17.3%) fetuses demonstrated genetic abnormalities at least in one testing modality (Figure 2, Tables 2 and 3). In 37 cases, all three testing modalities (karyotype, CMA, and ES) were performed. Among the cases with abnormal findings and tested by all three methods (*n* = 9), two CNVs in one fetus, detected by CMA, were also identified by ES‐based CNV calling (case #13). In 8 cases, ES showed a P/LP or a VUS, whereas CMA was normal. Focusing on ES results, 14 out of 66 fetuses (21.21%) revealed a total of 16 abnormal variants, including three variants of uncertain significance (VUS) in the *TBXT* gene (cases 4,9,10), and a VUS in the *MSX2* gene (Case 12). In 10 of 66 fetuses (15.15%), one or more pathogenic (P) or likely pathogenic (LP) variants have been found. In fetus 19, three different P or LP variants were detected, including one with a potential association with SD, and others with no known association. Of the detected variants, some have a known or potential association with SD, while others have no known association. Some of these non‐associated variants have well‐established clinical management paths, and targeted interventions may alter the natural history of the disease. In contrast, others worsen the prognosis, have implications for neurodevelopment, and have no available disease‐modifying treatments. Causative (pathogenic or likely pathogenic) variants for SD or other forms of NTD were not detected. A comprehensive summary of all genetic findings is provided in Tables 2, 3 and 4, and Supplementary Material (Table S1) as well as in Figure 3.

**TABLE 2:** Abnormal cytogenomic findings.

Case	Chromosome analysis ‐ FISH	CMA result size in Mb	CMA_ disease (OMIM)	CMA_ classification	Zygosity and inheritance
1	46,XX	arr[hg19]15q13.2q13.3(30940398_32515681)x1 1,6 Mb	Chromosome 15q13.3 microdeletion syndrome (612001)	Class 5, P	hz, I(mat)
2	46,XX	arr[hg19]22q11.21(20733667_21462353)x1 729 kb	22q11.2 microdeletion—Central B/C‐D	Class 4, LP	hz, I(pat)
3	mos 47,XY+20[5]/46, XY[35]	NP		NA	dn
5	46,XY	arr[hg19]17q25.3(76495050_81047565)x3,19p13.3(267039_782854)x1 4,6 Mb/0,5 Mb	^a^	Class 4, LP	hz, dn
13	46,XY,der(3)del(3) (p26.1)dup(3) (q29,q25.1)	arr[hg19]3p26.3p26.1(61495_6734031)x1,3q25.1q29(152022890_197838262)x3 6.7 Mb/44,8 Mb	^b^	Class 5 P	hz, dn
14	46,XX,t(1;16) (p34.1;q22).ish t(1;16) (wcp1+, CEB108/T‐, 16qTEL013+, wcp1+, 16pTEL05+, 16qTEL013‐,CEB108/T7+, wcp1+)	NP		NA	I(pat)
16	46,XY.ish del(22) (q11.2q11.2) (HIRA‐)	NP	DiGeorge syndrome (188400)	Class 5, P	hz, dn

*Note:* Karyotype and chromosomal microarray (CMA) abnormal findings are listed. Each row corresponds to a single patient.

Abbreviations: CMA, chromosomal microarray analysis; dn, de novo; F, female; hz, heterozygouse; I, inherited; M, male; mat, meternally inherited; NA, not available; NP, not performed; pat, paternally inherited.

^a^ Disease—causing OMIM Genes (a) in the deletion [19p13.3(267039_782854)x1]: BSG (109480), HCN2 (602781), POLRMT (601778b); (b) in the duplication [17q25.3(76495050_81047565)]: DNAH17 (610061), CANT1 (613165), CBX2 (602770), CCDC40 (613799), GAA (606800), EIF4A3 (608546), CARD14 (607211), SGSH (605270), RNF213 (613768), NPTX1 (602367), NDUFAF8 (618461), ACTG1 (102560), FSCN2 (607643), PDE6G (180073), MRPL12 (602375), SLC25A10 (606794).

^b^ Disease—causing OMIM Genes (a) in the deletion [3p26.3p26.1(61495_6734031)x1]: TRNT1 (612907), CRB*N* (609262), SUMF1 (607939), ITPR1 (147265); Omim‐Disease genes; (b) in the duplication [3q25.1q29(152022890_197838262)x3]: MME (120520), PLCH1 (612835), SLC33A1 (603690), GFM1 (606639), RSRC1 (6133529), B3GALT3 (603094), IFT80 (611177), SI (609845), BCHE (177400), PDCD10 (609118), SERPINI1 (602445), TERC (602322), SAMD7 (6200493), MECOM (165215), CLDN11 (601326), SLC7A14 (615720), SLC2A2 (138160), TNIK (610005), PLD1 (602382), GHSR (601898), SPATA16 (609856), NLGN1 (600568), TBL1XR1 (608628), PIK3CA (171834), GNB4 (610863), CCDC39 (613798), FXR1 (600819), SOX2 (184429), MCCC1 (609010), KLHL24 (611295), YEATS2 (613373), EIF2B5 (603945), DVL3 (601368), AP2M1 (601024), ALG3 (608750), EIF4G1 (600495), CLCN2 (600570), THPO (600044).

^a^^,^^b^: List of genes from UCSC Genome Browser (last access 6 May 2025), only dark green OMIM Genes (disease‐causing) have been listed above.

**TABLE 3:** Abnormal exome sequencing (ES) findings.

Case	ES_performance	ES_ resut	ES_ ACMG classification	ES_Gene known disease (OMIM), MOI
4	Singleton	c.245T>C, p.(Leu82Pro), hz, *TBXT* ENST00000296946	Class 3, VUS	Neural tube defects, susceptibility to (182940), AD
6	Trio	c.492del, p.(Asp1643IIefsTer35), hz, dn, *KDM3B* ENST00000314358	Class 4, LP	Diets‐Jongmans syndrome (618846), AD
7	Duo	c.58C>T, p.(Gln20Ter), hz, I, *ACVRL1* NM_000020.3	Class 5, P	Telangiectasia, hereditary hemorrhagic, type 2 (600376), AD
8	Trio	c.1985G>T, p.(Gly682Val), hz, dn, *SMC3*	Class 4, LP	Cornelia de lange syndrome 3 (610759), AD
9	Trio	c.302G>T, p.(Arg101Leu), hz, I, *TBXT* ENST00000296946	Class 3, VUS	Neural tube defects, susceptibility to (182940), AD
10	Duo	c.811C>T, p.(Pro271Ser), hz, I, *TBXT* ENST00000296946	Class 3, VUS	Neural tube defects, susceptibility to (182940), AD
11^c^	Trio	c.7099G>A; p.(Ala2367Thr), hz, I, *RYR1* NM_000540.2	Class 4, LP	King‐denborough syndrome (619542), AD; Congenital myopathy 1A, autosomal dominant, with susceptibility to malignant hyperthermia (117000), AD; malignant hyperthermia susceptibility 1 (145600), AD
12	Trio	c.2T>C, p.(Ala2_Met53del), hz, I, *MSX2* ENST00000239243	Class 3, VUS	Craniosynostosis 2 (604757), AD; Parietal foramina 1 (16850), AD; Parietal foramina with cleidocranial dysplasia (16855), AD
13	Trio	del(3) (p26.3‐p26.1) chr3:238239.5257653, hz^a^ dup(3) (q25.1–29) chr3:152017932–197765588, hz^b^	Class 5, P Class 5, P	Clinically significant chromosome 3 imbalances: Del involve critical diseases genes *CRBN* and *CNTN4*; dup involves critical region for 3q trisomy syndrome
15	Trio	c.563C>T, p.(Ser188Phe), hz, I *G6PD* NM_001360016.2	Class 5, P	Anemia, congenital, nonspherocytic hemolytic, 1, G6PD deficient (300908), X‐l
16^d^	Singleton	del(22) (q11.21) chr22:g.18836384–21563465del, hz	Class 5, P	DiGeorge syndrome (188400)
17	Trio	c.378_379dup, *p*(Leu127ArgfsTer8), hz, dn *SIN3A* ENST00000360439	Class 5, P	Witteveen‐kolk syndrome (613406), AD
18	Trio	c.1549_1552del, p.(Ser517ProfsTer23), hz, dn *CUL3* ENST00000264414	Class 5, P	Neurodevelopmental disorder with or without autism or seizures (619239), AD; pseudohypoaldosteronism, type IIE (614496), AD
19	Trio	c.1025A>G, p.(Lys342Arg), hz, dn *TGFBR1* NM_004612.4	Class 4, LP	Loeys‐dietz syndrome 1 (609192), AD
19	Trio	c.2080A>G p.(Met694Val), chz (allele 1) *MEFV* NM_000243.5	Class 5, P	Familial mediterranean fever (134610), AD; neutrophilic dermatosis, acute febrile (608068), AD
19	Trio	c.2230G>T, p.(Ala7444Ser), chz (allele 2) *MEFV* NM_000243.5	Class 4, LP	Familial mediterranean fever (134610), AD; neutrophilic dermatosis, acute febrile (608068), AD

*Note:* ES abnormal findings are listed, regardless of the clinical significance (includes potentially related and unrelated findings to neural tube defect). Results are reported according to the GRCh37/hg19 genome annotation. Each row corresponds to a single patient result.

Abbreviations: ES, exome sequencing; chz, compound heterozygous; dn, de novo; hz, heterozygous, I, inherited by a parent; LP, likely pathogenic; MOI, model of inheritance; NP, not performed; P, pathogenic, VUS, variant of uncertain significance, X‐l, X‐linked.

^a^ Terminal 3p deletion, size 5 Mb, including 13 protein‐coding genes.

^b^ Terminal duplication 3q, size 46 Mb, including at least 220 protein‐coding genes.

^c^ The *RYR1* variant was identified as a secondary finding unrelated to the neural tube defect. While the reporting laboratory classified it as likely pathogenic (class 4), ClinVar entries list the variant as VUS.

^d^ CMA was not performed as it is not covered by health insurance in Germany. Private insurance approved ES, but not CMA, after a delay; DiGeorge syndrome was thus diagnosed by ES.

**TABLE 4:** Fetal phenotypes, turn‐around time for genetic tests, and pregnancy outcomes.

Case	SD type	BMI (pre preg)	Mat. age	Other US findings (HPO)	Gestational age at diagnosis (weeks)	Gestational age at CMA result (weeks)	Gestational age at ES result (weeks)	Gestational age at surgery (weeks)	Genetic result influenced parental decision	Outcome
1	MS	38	24	No	23.14	27.0			No	TOP
2	MS	24	25	No	25.28	27.71		26.71	No (result available after surgery)	Surgery pre
3	MMC	38	32	Perianal appendage (caudal appendage HP:0002825)	20.14	21.86				Lost to FUP
4	MMC	20	32	No	21.86	25.71	24.86		No	TOP
5	MMC	27	31	No	16.71	19.86			Yes	TOP
6	CD	25	37	No	21.57		25.29		No	Surgery post
7	MS	18	31	No	23.29	28.0	28.43		No	TOP
8	MMC	24	36	Malalignment ventricular septal defect HP:0001629, low‐set ears HP:0000369, single umbilical artery HP:0001195	19.43		21.29		Yes	TOP > surgery pre
9	MMC	36	24	No	21.14		25.43	25.57	No	Surgery pre
10	MMC	18	26	No	20.71	23.57	24.29		No	TOP
11	MMC	29	28	No	22.14		22.86		No	Surgery post
12	MMC	22	30	No	17.28		22.57		No	TOP
13	MMC	25	24	No	21.0	30.43	25.43		No	TOP
14	MMC	21	31	No	25.14		28.29	26.0	No	Surgery pre
15	CD	26	31	No	21.57		23.86		No	Surgery post
16	CD	22	34	No	17.71		29.29		Yes	TOP > surgery post
17	MMC	26	36	No	22.14	28.0	24.86		Yes	TOP > surgery pre
18	MS	19	30	No	19.29		23.71		Yes	TOP > surgery pre
19	MS	25	39	No	21.43		28.29		No	TOP

*Note:* The table indicates fetal phenotype: SD type, other US findings, and fetal age at the diagnosis; selected maternal characteristics: BMI and age; ages at CMA and ES results; age at surgery, genetic result implication for parental decision, and outcome. Each row corresponds to a case with an abnormal genetic finding.

Abbreviations: CD, closed dysraphism; MMC, Meningomyelocele; MS, Myeloschisis; SUA, single umbilical artery; TOP, termination of pregnancy, surgery pre: prenatal repair, surgery post: postnatal repair; VSD, ventricle septum defect.

### 3.5 Turnaround Time and Impact of Genetic Results on Clinical Management

The median gestational age at receipt of CMA results was 27.0 weeks (23.6–28.0), whereas ES results became available at a median gestational age of 24.9 weeks (23.8–26.9). The median turnaround time from diagnosis to availability of results was 3.9 weeks (2.97–4.99) for CMA and 3.7 weeks (2.9–4.9) for ES and varied substantially across cases (Table 4). The sometimes‐considerable delay in the availability of results was not attributable to the analytical method itself, for which the average technical turnaround time was approximately 2–3 weeks. Instead, the primary contributing factors were logistical constraints and delays associated with the notification and confirmation of cost coverage by health insurance providers. Four out of 66 (6.1%) patients revised their initial therapeutic decisions based on ES findings. Three patients initially opted for prenatal fetal surgery but subsequently elected termination of pregnancy (TOP) after receiving abnormal genetic results. All genetic results in these cases (cases 8, 17, 18) were available before surgical intervention. One patient with a closed SD (Case 16), planned for postnatal surgery, underwent only karyotype and ES, due to private insurance constraints that excluded CMA coverage. After a lengthy approval process, ES results were eventually obtained at 29.3 weeks, and subsequent detailed genetic counseling resulted in termination of pregnancy (TOP). Another case (Case 2), presented at 25.3 weeks, received abnormal CMA results post‐fetal surgery; this pregnancy was continued. Parental testing identified the father as a carrier of the same 22q11.2 microdeletion (Table 4).

### 3.6 Anatomic Anomalies and Association With Genetic Findings

Within the entire cohort (*n* = 150), eight fetuses exhibited non‐severe, non‐associated anatomic anomalies. These anomalies included hydronephrosis, tricuspid insufficiency, corpus callosum lipoma, malalignment ventricular septal defect (VSD), and low‐set ears, muscular VSD, perianal appendage, and single umbilical artery. Among fetuses with genetic abnormalities (*n* = 19), 2 (10.5%) presented at least one additional non‐associated malformation, compared to 3 (3.3%) in those without genetic anomalies (*n* = 91). This difference was not statistically significant (*p* = 0.2). Case 13 exhibited isolated open SD on ultrasound without additional major structural anomalies, an atypical yet documented presentation in the context of large chromosomal imbalances involving chromosome 3. Parental testing confirmed both CNVs to be de novo. The pregnancy was terminated at an external institution, and post‐mortem data were not available.

## 4 Discussion

In this study, we analyzed a cohort of 150 fetuses diagnosed with spinal dysraphism to explore underlying genetic causes and their effect on parental decision‐making. Invasive testing was performed in 110 cases. Genetic testing, employing karyotype, CMA and ES revealed abnormal findings in 17.3%. In cases where ES was performed, 15.2% showed one or more pathogenic or likely pathogenic variants. Eight out of 37 cases, in which all three modalities were applied, showed a pathogenic or likely pathogenic variant that was only detected by ES. The identified variants represent a genetically heterogeneous spectrum and have been categorized into two major groups according to the clinical role: (1) findings with previously reported or plausible mechanistic involvement in SD; and (2) findings unrelated to SD and associated with significant prognosis modification (conditions with severe post‐natal phenotype, including neurodevelopmental impairment) or leading to medical actionability (conditions with a well‐described management, treatment, reproductive counseling). In four cases, identified variants led to a change in parental decision, resulting in termination of pregnancy instead of pre‐ or postnatal surgery. These results highlight the clinical impact of comprehensive genetic testing in fetal spinal dysraphism, regardless of the etiologic yield.

### 4.1 Detection Rate of Causative Variants on CMA and ES

Causative variants were not detected. This may be partly due to the small sample size and is consistent with the multifactorial etiology of NTDs and confirms that, even after comprehensive CMA and ES analyses, no single genetic variant currently explains the occurrence of NTDs. This finding is relevant for family counseling and supports the need for integrative studies addressing the interplay between genetic, epigenetic, and environmental factors in neural tube development.

### 4.2 Numerical or Structural Chromosomal Abnormalities Associated With NTDs

Numerical or structural chromosomal abnormalities such as mosaic trisomy 20 (Case 3), 22q11.2 deletions (cases 2 and 16), and large genomic rearrangements involving chromosomes 3 and 17/19 (cases 5 and 13) have previously been associated with various NTD phenotypes [8, 39, 40, 41]. While loss of *CRKL* within the recurrent 22q11.2 deletion is known to confer increased risk for meningomyelocele, many of the other observed chromosomal abnormalities likely reflect broader genomic instability or dosage‐sensitive effects, in which the underlying mechanisms remain to be elucidated [42].

### 4.3 Selected Variants in Genes With Established, Candidate, or Putative Roles in NTDs

Notably, *TBXT* emerged as a recurrent candidate gene in three cases (4, 9, 10), aligning with its established role in mesodermal specification and axial elongation during embryogenesis. Functional studies in murine and zebrafish models show that loss‐of‐function mutations in *Brachyury* (mouse homolog of *TBXT*) lead to severe notochordal and somitic defects, consistent with observed phenotypes including myelomeningocele and closed spinal dysraphism [43, 44, 45]. Similarly, *MSX2*, a homeobox transcriptional repressor involved in craniofacial and neural crest development, was implicated in Case 12 and has previously been reported in association with exencephaly and occipital encephalocele [44, 45, 46, 47]. Mouse models support its role in neural tube closure, possibly mediated through BMP‐dependent signaling pathways [44, 45] (Table S1).

Additional candidate genes include *SMC3* (Case 8), encoding a subunit of the evolutionary conserved multimeric cohesin complex and whose dysfunction can lead to developmental abnormalities, including those affecting the central nervous system, *SIN3A* (Case 17), which encodes a transcriptional regulator involved in chromatin remodeling, *CUL3* (Case 19), implicated in cortical lamination and neural migration through ubiquitin‐proteasome‐mediated pathways [48]. *KDM3B* (Case 6), a histone demethylase involved in transcriptional regulation, has been shown in murine models to be critical for early development and cerebellar function. These genes' involvement in NTDs and neurodevelopmental processes warrants further investigation.

For other disease genes, an indirect role in the etiopathogenesis of NTDs may be proposed, even in the absence of definitive supporting evidence. *ACVRL1*, primarily known for its role in angiogenesis and its association with Hereditary Hemorrhagic Telangiectasia type 2 (HHT2), encodes a TGF‐β type I receptor involved in the TGF‐β signaling pathway, which regulates cell proliferation, differentiation, and apoptosis—key processes during neural tube closure [49, 50]. While *ACVRL1* has not previously been directly linked to NTDs, other components of the TGF‐β pathway (e.g., SMADs, BMPRs) have been implicated in NTDs in animal models and in human fetal tissue, where altered expression of SMAD proteins has been observed at the sites of failed neural tube closure [51].

Finally, other variants (e.g., *G6PD*, *TGFBR1*, *MEFV*, *RYR1*) lacked a phenotypic association with NTDs but warrant dedicated clinical management and familial counseling.

### 4.4 Added Diagnostic Value of ES Beyond Karyotype and CMA

Among fetuses exhibiting no detectable structural anomalies on prenatal imaging, ES identifies genetic alterations in an estimated 2%–3% of cases [52, 53, 54, 55]. Depending on the organ system and the number of organ systems involved, ES increases the diagnostic yield by over 30% in structurally abnormal fetuses when chromosomal microarray or karyotyping is non‐diagnostic [36, 56]. This has also been demonstrated in prenatal cohorts with central nervous system (CNS) anomalies [57, 58, 59, 60, 61, 62, 63]. A recent trio‐based postnatal study reported a 22.3% rate of de novo variants in individuals with myelomeningocele, with 28% of these variants deemed pathogenic, a figure consistent with the prenatal detection rate in our cohort [64]. While prenatal studies have primarily focused on conditions such as agenesis or dysgenesis of the corpus callosum, ventriculomegaly and gyration anomalies, the present study is, to our knowledge, the first to systematically evaluate the role of prenatal ES in fetuses with SD. The findings of our study are consistent with previously published data and suggest that ES offers an incremental value compared with conventional karyotyping and CMA. This supports the inclusion of ES in the standard diagnostic algorithm for SD before surgery [5, 65].

While concerns were raised regarding the routine use of preoperative ES, citing potential delays and limited yield, current sequencing turnaround times of approximately two to 3 weeks are compatible with the critical windows for fetal surgical intervention in a non‐emergency setting like fetal SD surgery [66]. Some outliers within our cohort were not attributable to technical limitations but rather to reimbursement issues, which resulted in clinically relevant delays in the diagnosis. Timely detection of SD by prenatal ultrasound is critical to ensure sufficient time for genetic evaluation and subsequent clinical decision‐making. In four cases of our cohort, the genetic diagnosis led to a change in prenatal management. In three cases, parents opted for termination instead of prenatal surgery, and ES results were available before surgery. In one case, planned for postnatal intervention, termination was also chosen. All diagnoses were established by ES; in Case 17, CMA was not performed due to reimbursement issues but could have led to the diagnosis. Even when results become available only after fetal surgery, they may still contribute to optimizing postnatal management and enable appropriate follow‐up planning in continuing pregnancies. Given the procedural risks and resource intensity associated with fetal surgery, the pre‐operative identification of underlying genetic conditions should be regarded as a prerequisite for optimal case selection and counseling [67].

Nevertheless, significant interpretative challenges persist. These include the potential role of VUS, variable expressivity, incomplete penetrance, and limited genotype–phenotype correlations of variants. Moreover, as all analyzed samples were obtained from amniotic fluid, somatic mosaicism confined to neural tissue cannot be excluded [68].

### 4.5 Strengths and Limitations

This study represents the first focused analysis of ES in a well‐characterized prenatal cohort with spinal dysraphism. A principal strength lies in the integration of detailed fetal phenotyping with high‐resolution genomic interrogation, allowing both the elucidation of potentially causative pathways and the identification of clinically relevant secondary findings. The inclusion of variants across a spectrum of genomic alterations—from well‐established CNVs to emerging candidate genes—provides novel insights into the etiological heterogeneity of spinal dysraphism. Furthermore, the demonstration of feasible turnaround times for ES (2–3 weeks) supports its utility within existing clinical decision‐making frameworks for prenatal and perinatal care and therapy.

Limitations include the retrospective design, which may have introduced selection bias. Trio‐based ES was not uniformly performed, which may have constrained variant interpretation and filtering strategies. In addition, the absence of systematic phenotypic assessment of parental carriers restricted the evaluation of variant pathogenicity. Lastly, the exclusive use of amniotic fluid as a DNA source limits the detection of tissue‐specific mosaicism that may be present in affected neural structures.

## 5 Conclusion

Based on our current knowledge and the considerations outlined above, the inclusion of trio‐based ES or potentially whole genome sequencing in the diagnostic work‐up of fetuses with SD may be increasingly considered as a relevant adjunct to standard testing and has an impact on parental decision‐making, even though the detection rate of causative molecular findings is low. Further prospective studies are needed to determine the optimal integration of this approach into clinical practice, the choice of bioinformatic algorithms for variant filtering and reporting, as well as its impact on prenatal surgical decision‐making, pregnancy outcome, and long‐term neurodevelopmental outcomes.

## Funding

The authors have nothing to report.

## Ethics Statement

Initial ethical approval for this study was obtained from the University Hospital Institutional Review Board in Giessen (IRB approval AZ 161/20) and Marburg (IRB approval AZ 23–280 BO).

## Consent

Because of the retrospective nature of the study, informed consent from the patients was waived as the study analyzed anonymized clinical patient data.

## Conflicts of Interest

The authors declare that they have no conflict of interest. At the moment of submission, AF is an external consultant for Menarini Silicon Biosystems S.p.A., working on the cbNIPT project.

## Supporting information


**Table S1:** Abnormal molecular findings with known or potential association with SD.

## Data Availability

The data used to support this study's findings are available from the corresponding author upon reasonable request.
