# Supplementary material for: Uncovering the Genetic Landscape of Spinal Dysraphism: A Retrospective Analysis of 150 Fetal Cases
Source: Prenat Diagn. 2025 Dec 12;46(5-6):849–61. doi: 10.1002/pd.70037 (PMC13170066; doi:10.1002/pd.70037)
Supplement: Supplementary file 1 — Table S1: Abnormal molecular findings with known or potential association with SD. [file PD-46-849-s001.docx]

**Table S1 –** **Abnormal molecular findings with known or potential association with SD**

| **Case(s)** | **Result** | **Phenotype(s) (OMIM/ORPHA), MOI** | **Penetrance \| clinical expressivity** | **NTD among HPO Associations *** | **Any association with NTD (type)** | **Protein: involvement in neural tube development** | **Protein: Functional module** | **Major references** |
| --- | --- | --- | --- | --- | --- | --- | --- | --- |
| 2 | 22q11.2 microdeletion – Central B/C-D | 22q11.2 microdeletion – Central B/C-D, AD | Incomplete \| variable | No | Yes (MMC) | Unknown – limited evidence for biallelic involvement in SNAP29 | NA | PMID: [25123577](https://pubmed.ncbi.nlm.nih.gov/25123577/)  PMID: 21073448  PMID: 15968592 |
| 3 | Mosaic trisomy 20 in amniotic fluid | Mosaic trisomy 20 syndrome (ORPHA:1724) | NA \| variable | [HP:0002143](https://hpo.jax.org/browse/term/HP:0002143) Abnormal spinal cord morphology | Yes (EC, AN) | Unknown | NA | PMID: 18182338 |
| 4, 9, 10 | *TBXT* | Neural tube defects, susceptibility to (182940), AD  Sacral agenesis with vertebral anomalies (615709), AR | Incomplete \| variable | [HP:0003298](https://hpo.jax.org/browse/term/HP:0003298) Spina bifida occulta  [HP:0002475](https://hpo.jax.org/browse/term/HP:0002475) Myelomeningocele | Yes | Brachyury, T-box transcription factor T: Mesoderm formation and differentiation, notochord development, somitogenesis and neural tube formation | Mesoderm Specification and Axis Elongation Pathway | PMID: 20477849; PMID: 37751845 |
| 8 | *SMC3* | Cornelia de Lange syndrome (CdL) 3 (610759), AD | High, incomplete \| variable | No | Thoracic meningogele in CdL | Structural Maintenance Of Chromosomes 3: Not directly.  Involved in *fundamental cellular processes* | Cohesin Complex Pathway | PMID: 28408410  PMID: 20375878 |
| 12 | *MSX2* | Craniosynostosis 2 (604757), AD;  Parietal foramina 1 (16850), AD;  Parietal foramina with cleidocranial dysplasia (16855), AD | High, incomplete \| extremely variable | [HP:0002084](https://hpo.jax.org/browse/term/HP:0002084) Encephalocele  [HP:0002085](https://hpo.jax.org/browse/term/HP:0002085)  Occipital encephalocele [HP:0002475](https://hpo.jax.org/browse/term/HP:0002475) Myelomeningocele | Yes (EX) | MSH homeobox*: Developmental transcription regulation and neural crest development* | Developmental Gene Regulatory Network (BMP-Dependent) | PMID: 11320527, PMID: 21362336  PMID: 16221730; PMID: 23425387; |
| 13 | Deletion 3p and duplication 3q | Various disease-genes involved °° | NA | NA | Yes | Unknown | NA | PMID 18438967;  PMID: 22212322 |
| 16 | 22q11.2 deletion syndrome | DiGeorge syndrome (188400) | Incomplete \| variable | [HP:0002435](https://hpo.jax.org/browse/term/HP:0002435) Meningocele  [HP:0002414](https://hpo.jax.org/browse/term/HP:0002414) Spina bifida | Yes (MMC, sacral MC) | Unknown – limited evidence for biallelic involvement in SNAP29 | NA | PMID: 38696583  PMID: 25123577  PMID: 21073448  PMID: 15968592  PMID: 18182338 |

CMA: chromosomal microarray, K: karyotype, ES: exome sequencing; AN: anencephaly; EC: encephalocele; EX: exencephaly; MC: meningocele; MMC: myelomeningocele, NA: not available

*According to Human Phenotype Ontology (https://hpo.jax.org/ , last assessment 04/26/2025)
